# Littré’s Hernia: A Rare Intraoperative Finding

**DOI:** 10.7759/cureus.11065

**Published:** 2020-10-20

**Authors:** Ahmad Usman, Muhammad Humayoun Rashid, Umar Ghaffar, Umar Farooque, Ayesha Shabbir

**Affiliations:** 1 General Surgery, Nishtar Medical University and Hospital, Multan, PAK; 2 Cardiology, Ch. Pervaiz Elahi Institute of Cardiology, Multan, PAK; 3 Internal Medicine, Nishtar Medical University and Hospital, Multan, PAK; 4 Neurology, Dow University of Health Sciences, Karachi, PAK

**Keywords:** littre's hernia, meckel's diverticulum, strangulation, obstruction

## Abstract

Littré’s hernia (LH) is a rare clinical entity defined by the presence of Meckel’s diverticulum (MD) within a hernial sac. Although MD is the most common congenital abnormality of the intestinal tract, most of its cases remain asymptomatic. It may, however, manifest itself in the form of multiple complications. One of its rare complications is LH, which is reported occurring in a mere 1% of all MD cases. The anamneses of LH are like any other hernia containing the gut, making its preoperative diagnosis unlikely. We present herein a case of a 16-year-old boy with an incarcerated LH at the inguinal region, which was successfully treated by wedge resection of the diverticulum followed by hernia repair.

## Introduction

Meckel’s diverticulum (MD) is the most common congenital anomaly of the intestinal tract with a reported incidence of 0.6%-4% [1]. It is a true diverticulum containing all three layers of the gut, usually located 30-90 cm from the ileocecal junction at the anti-mesenteric border of the ileum. It is known to develop from the incomplete resorption of the omphaloenteric duct through which the fetal midgut communicates with the umbilical vesicle until the fifth week of gestation. Its mucosa may contain ectopic gastric (in 23%-50% cases), pancreatic (in 5%-16% cases), or infrequently colonic tissue [2]. MD generally remains clinically silent. Mostly, it is a mere fortuitous discovery during a laparotomy performed for another reason or rarely it may incidentally be diagnosed by imaging studies. Nevertheless, it can present with multiple complications as well, including intestinal obstruction, bleeding, intussusception, perforation, etc. One of its rare complications is when MD protrudes into the hernia sac, a condition called Littré’s hernia (LH).

LH is an extremely rare condition, found in only 1% of all cases of MD. Over the past 300 years, less than 50 cases have been described in the literature [3]. The actual incidence of LH is hitherto unknown but has been reported to be only 0.09% of incarcerated or strangulated hernias [4]. Historically, this condition got its name after Alexis de Littré, a French surgeon, who in the 1700s reported his findings of this condition in two of his patients. Later, Reinke established the term in 1841, when he defined it as ‘Meckel’s diverticulum inside any hernial sac’ [5].

LH can present as an inguinal, femoral, or umbilical hernia with symptoms and complications like any other hernia with the small intestine in its contents. The herniating part of the gut can get obstructed, incarcerated, or even strangulated just like other hernias [4,6]. Hence, the diagnosis is established only intraoperatively on the exploration of the hernial sac. We report herein a case of an incarcerated LH, which to our knowledge will be this first reported case of LH from Pakistan, with the purpose to further enrich the current literature for the better management of this rare entity.

## Case presentation

**Figure 1:**
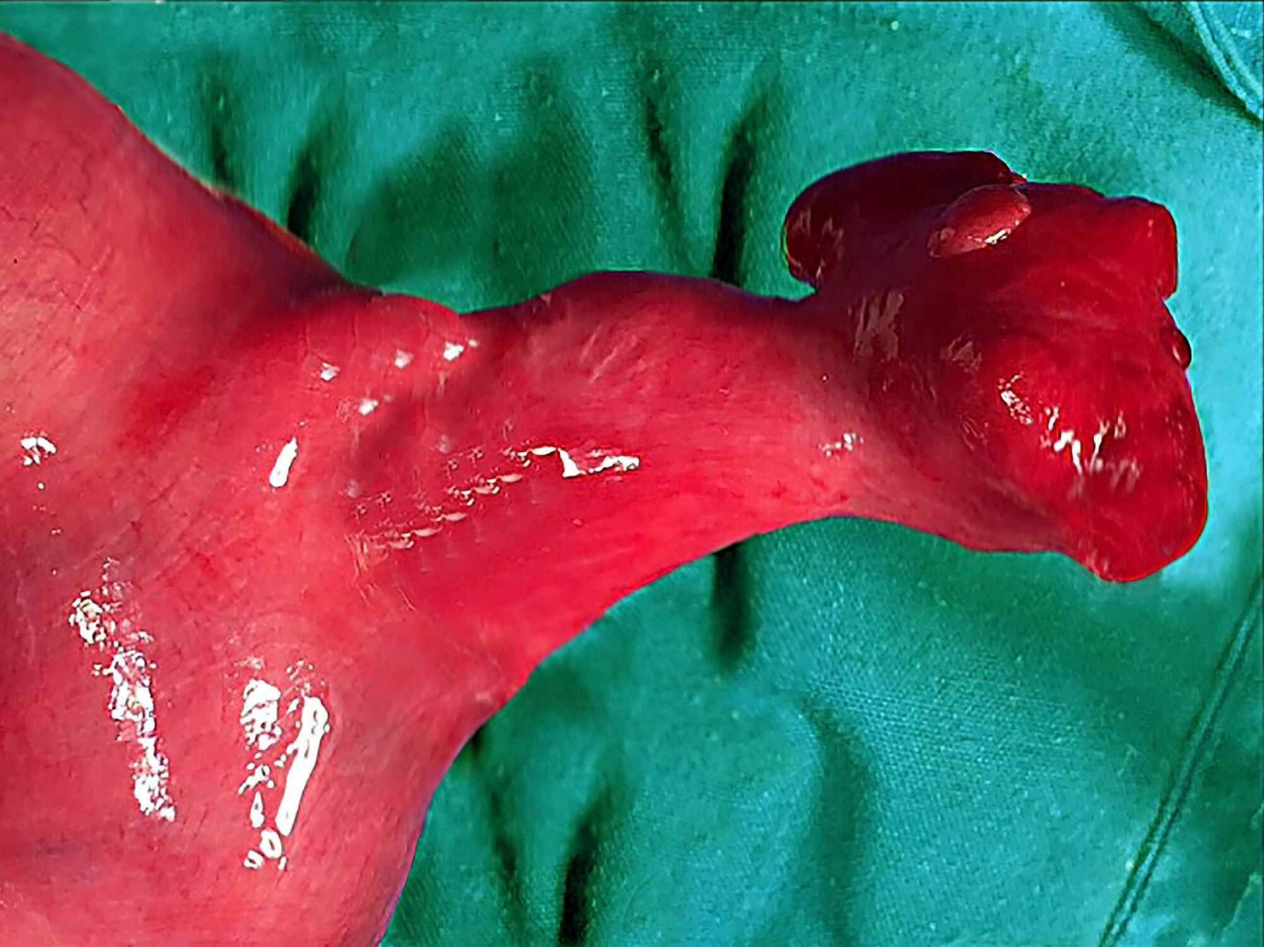
Intraoperative view of Meckel’s diverticulum

A 16-year-old male patient presented to the Accident and Emergency department of Nishtar Medical Hospital, Multan, with a two-hour history of severe pain of abrupt onset in his left groin associated with nausea. He gave no history of any lump or hernia in this region in the past. Except for mild tachycardia, the patient was stable hemodynamically. The local examination revealed an erythematous, tender, and warm swelling of 3 x 3 cm in the left inguinoscrotal region, which was irreducible and had no cough impulse. The abdomen was soft, non-tender showing no signs of peritonitis. The preoperative biochemical and hematological investigations were in the normal range except for mild leukocytosis (13.2 x 10^3^/L) with neutrophilia. A diagnosis of incarcerated left inguinal hernia with possible strangulation of its contents was made. The patient was immediately operated for exploration of the inguinal canal under general anesthesia via para-inguinal approach. Exploration of the hernial sac revealed a 3-cm-long incarcerated non-gangrenous Meckel’s diverticulum in it. The obstruction was released by widening the deep inguinal ring and the ileal loops were delivered into the wound to exclude other pathologies. The diverticulum and ileum regained their normal color and blood flow (Figure 1).

A decision of wedge resection of the diverticulum was made. The rest of the bowel was reduced back into the abdomen followed by repair of the inguinal wall by the Lichtenstein technique. The post-operative period remained uneventful and the patient was discharged on the fourth postoperative day. He was followed up on an outdoor basis with no complications seen after two months. The histopathological report of the resected diverticulum showed ileal mucosa with ischemic changes and no ectopic tissue.

## Discussion

MD is a common congenital anomaly of the gut. The description of a typical MD can be given by the ‘rule of 2’ according to which MD is seen in 2% of the population, is usually diagnosed under 2 years of age, measures 2 inches in size and 2 cm in diameter, lies 2 feet proximal to the ileocecal junction, is 2 times frequent in males and is symptomatic in 2% of the patients [7,8]. Although 90% of the cases are asymptomatic, yet it can cause a multitude of complications, the most common of which is the intestinal obstruction as seen in our case. Park et al. reviewed the data of 1476 patients with MD and found that the most common clinical presentation of MD in children was obstruction and in adults, bleeding [9]. Another review done on 600 patients of MD by Ymaguchi et al. also showed the most common complication to be obstruction, found in 36.5% of cases [10]. Obstruction can occur through various mechanisms. MD may act as the leading point for intussusception or may cause volvulus of gut around the band by which it remains attached to the umbilicus. The diverticulum along with the gut may also get entrapped within the mesentery or get strangulated in an internal or external hernia [6]. It is when the diverticulum protrudes into a hernia, we call the condition as Littré’s hernia.

Groin hernias are prone to incarceration or strangulation, and their contents also vary. Based on their contents, three types of hernias are often described: Littré’s hernia (contains MD), Amyand hernia (contains appendix), and Richter’s hernia (contains the small intestine’s antimesenteric part). Infrequently, the ovary or fallopian tubes may also be found in hernial sacs [11]. There are no characteristic diagnostic clinical symptoms or signs to differentiate LH from other hernias. However, a history of rectal bleed, incomplete manual reduction of the hernia, and fecal fistulas must alert the surgeon of the possibility of LH [12,13]. Ultrasonography, CT scan, scintigraphy, and Technetium-99m scan when used in combination may help in preoperative diagnosis, as suggested by Augestad et al. and Levy and Hobbs in their respective studies [14,15]. Contrary to this, Messina et al. [5] and Luengas et al. [16] in their studies concluded that it may not be possible to diagnose MD protruding into a hernia sac even with the aid of multiple imaging modalities. Additionally, in clinical settings like ours, the latest modalities like Technetium scans and scintigraphy facilities are not readily available. The poor socioeconomic status of the masses also comes into play when patients cannot afford expensive investigations. Surgeons, therefore, have to rely on bedside examination and basic investigations, making a preoperative diagnosis of such rare cases almost impossible.

In our case, the patient presented with pain and mass in the groin. He showed no signs of mechanical intestinal obstruction clinically or on the abdominal radiograph despite the fact that his diverticulum was getting strangulated. One likely explanation for this can be the fact that in LH, initially only the diverticulum is compromised, and not the small intestine itself. The bowel, being free, does not give signs of intestinal obstruction. This resembles a typical presentation of Richter’s hernia in which the lumen of the intestine is only partially contained in the hernia, giving no signs of obstruction initially [17]. That’s why incarcerated LH symptoms are late in onset and less severe.

The management of MD depends on the clinical presentation. When it is symptomatic, the treatment is surgery. However, in asymptomatic cases, there is no consensus on the best mode of treatment, the reason being that it is not possible to gauge the risk of complications with an incidentally detected MD by intraoperative inspection or palpation. Some surgeons favor prophylactic resection of an incidentally encountered MD due to the potential for life-threatening complications that may develop later on, and secondly, because of the lower morbidity rates associated with the resection of a normal diverticulum compared to the resection of a pathological diverticulum [18]. Robijn et al. proposed a risk scoring system to be used as a guide in making the decision of resection or no resection of MD [19]. The currently available literature suggests the removal of all incidentally discovered diverticula if they meet the following criteria: age <50 years, male gender, diverticulum length >2 cm, diverticulum broad-based, fibrous bands attached to the diverticulum and histopathological examination showing ectopic or abnormal tissue [1].

Symptomatic cases of MD including LH are treated surgically by open or laparoscopic techniques. The simple wedge resection of the diverticulum is usually done. However, segmental ileal resection with end-to-end anastomosis may be necessary in complicated cases where ischemic inflammatory changes are reaching the base of the diverticulum, and when the diverticulum is broad-based with palpable heterotopic tissue [1,13].

We performed wedge resection of MD in our case because the diverticulum was longer than 2 cm, had a broad base and was ischemic. Ileal resection was not considered as the portion of the small gut that was incarcerated regained its blood flow with normalization of the color, and the diverticulum had no palpable mass. Although modern trends favor minimally invasive techniques, yet socioeconomic limitations made such an approach not feasible in our case.

## Conclusions

The presentation of Littré’s hernia is similar to any other hernia, making its preoperative diagnosis a big challenge. The probability of finding MD in a hernial sac must be kept in the surgeon’s mind especially while examining a pediatric patient with swelling at the inguinoscrotal region. A history of recurrent rectal bleed or abdominal pain with the presence of an incarcerated irreducible hernia may point to the presence of MD. Manual reduction maneuvers should be avoided in such incarcerated hernias. The MD present in Littré’s hernia should always be resected. Bowel resection should also be considered if the bowel segment bearing the diverticulum shows irreversible ischemic inflammatory changes.

Sound knowledge of the anatomical, clinical, radiological and pathological features of MD can aid in early diagnosis and timely intervention, precluding the risk of complications. Moreover, surgeons all over the globe must keenly highlight and report more cases of this rare condition to enrich the literature with better diagnostic and management plans for this rare condition.
